# Regulation of Skeletal Muscle Glucose Transport and Glucose Metabolism by Exercise Training

**DOI:** 10.3390/nu11102432

**Published:** 2019-10-12

**Authors:** Parker L. Evans, Shawna L. McMillin, Luke A. Weyrauch, Carol A. Witczak

**Affiliations:** 1 Department of Kinesiology, East Carolina University, Greenville, NC 27858, USA; evanspa17@students.ecu.edu (P.L.E.); mcmillins15@students.ecu.edu (S.L.M.); weyrauchl16@students.ecu.edu (L.A.W.); 2 Department of Physiology, Brody School of Medicine, East Carolina University, Greenville, NC 27834, USA; 3 East Carolina Diabetes & Obesity Institute, East Carolina University, Greenville, NC 27834, USA; 4 Department of Biochemistry & Molecular Biology, Brody School of Medicine, East Carolina University, Greenville, NC 27834, USA

**Keywords:** aerobic exercise, blood glucose, functional overload, GLUT, hexokinase, insulin resistance, resistance exercise, SGLT, type 2 diabetes, weightlifting

## Abstract

Aerobic exercise training and resistance exercise training are both well-known for their ability to improve human health; especially in individuals with type 2 diabetes. However, there are critical differences between these two main forms of exercise training and the adaptations that they induce in the body that may account for their beneficial effects. This article reviews the literature and highlights key gaps in our current understanding of the effects of aerobic and resistance exercise training on the regulation of systemic glucose homeostasis, skeletal muscle glucose transport and skeletal muscle glucose metabolism.

## 1. Introduction

Exercise training is defined as planned bouts of physical activity which repeatedly occur over a duration of time lasting from weeks to years. For the purposes of this review article, we have divided exercise training into two general categories: (1) Aerobic exercise training, which consists of weight bearing and non-weight bearing activities; and (2) resistance exercise training, which consists of weight bearing activities that act against an external load. Both types of training can be developed as progressive programs, which is defined as a planned increase in the duration, frequency, and/or intensity of the activity throughout the training period. The objective of this article was to review the literature on the effects of aerobic and resistance exercise training on the regulation of systemic glucose homeostasis, skeletal muscle glucose transport and glucose metabolism, and then to highlight gaps in our current understanding of these key adaptations. To accomplish this objective, we performed searches of the scientific literature utilizing Google Scholar, Highwire, PubMed, and Scopus to identify studies that contained the following keywords (alone and in combination): Aerobic exercise; aerobic training; blood glucose; cycling; glucose homeostasis; elastic band; exercise; exercise adaptations; exercise training; functional overload; glucose homeostasis; glucose metabolism; glucose transporter; glucose uptake; facilitative glucose transporter (GLUT); glycolysis; glycolytic flux; glycogen content; glycogen synthesis; hexosamine pathway; ladder climbing; overload; pentose phosphate pathway; resistance exercise; resistance training; resistance training adaptations; running; sodium-dependent glucose co-transporter (SGLT); skeletal muscle; sodium-dependent glucose co-transporter; swimming; synergist ablation; treadmill; type 2 diabetes; weight lifting; weight training; wheel cage. The identified articles were then divided into either the aerobic or resistance exercise training categories based on whether an external load was acted against during the exercise. Only studies using a 6-week training intervention or longer were selected for this review, with the exception of one article. The training period for this article was 3 weeks, and that information is clearly indicated in that section. We allowed this one exception because it highlighted a key gap in the current literature regarding the mechanism(s) underlying the effects of aerobic or resistance exercise training on muscle glucose transport.

## 2. Models of Aerobic and Resistance Exercise Training

Aerobic exercise training exists in a wide variety of forms, and the following list includes examples of aerobic exercises that are routinely performed by individuals in self-initiated aerobic training programs: aerobic classes, cycling, dancing, jumping jacks, jumping rope, rowing, running, skating, skiing, swimming and walking. In aerobic training research studies, the most common forms of exercise utilized in human subjects are treadmill running and cycle ergometer training [1,2,3,4], whereas in animal studies the most common form is treadmill running [5,6,7]. Resistance exercise training also exists in a variety of forms, and the following list includes examples of exercises that are routinely performed with weights or elastic bands by individuals in self-initiated resistance training programs: bicep curl, shoulder press, bench press, barbell squat, bent over row, and lateral band walk. In resistance training research studies, the most common form of exercise utilized with human subjects is a weight-bearing program involving the upper body, lower body and abdomen [8,9,10]. In contrast, in resistance training studies involving rodents the most common form of exercise is weighted ladder climbing [11,12,13,14]. While aerobic exercise training activities are naturally common in both humans and animals due to survival instincts (i.e., chasing down prey or running away from a predator), resistance exercise training activities (i.e., carrying an external load) are not. Thus, there are fewer studies that have investigated the physiological effects of resistance exercise training in animal models. To overcome this challenge, a surgical approach was developed that rapidly and reproducibly induces loading/functional overload in rodent skeletal muscle via the removal of one or more synergist muscles [15]. Importantly, numerous studies have shown that functional overload induces the same adaptations as resistance exercise training in skeletal muscle, including increases in muscle size and muscle strength [16,17,18,19,20,21,22,23].

## 3. Aerobic and Resistance Exercise Training-Induced Adaptations

Aerobic exercise training and resistance exercise training are both well-known for their ability to induce specific beneficial adaptations in the human body. For aerobic exercise training, the predominant adaptations are in the cardiorespiratory system and include: (1) A decrease in resting heart rate [24,25,26]; (2) a decrease in resting blood pressure [24,25,26]; and (3) an increase in maximal oxygen uptake (VO_2_ max) [27,28]. For resistance exercise training, the predominant adaptations are in the musculoskeletal system and include: (1) An increase in muscle mass [29,30,31]; (2) an increase in muscle strength [32]; and (3) an increase in bone density [33,34]. For a thorough review on the effects of aerobic and resistance training on these cardiovascular and musculoskeletal adaptations see the following article: [35]. In addition to these adaptations, both aerobic exercise training and resistance exercise training are well-known for their ability to restore systemic glucose homeostasis in individuals with the metabolic disease type 2 diabetes. In the following section on the regulation of systemic glucose homeostasis, we describe how blood glucose levels are regulated in a healthy state, and then review the current literature regarding how they are impacted by type 2 diabetes and exercise training.

## 4. Regulation of Systemic Glucose Homeostasis

### 4.1. Regulation in Healthy Individuals

In healthy individuals, systemic glucose homeostasis is tightly regulated to maintain fasted blood glucose levels at <100 mg/dl (<5.5 mM), glycated hemoglobin A1c (HbA1c) levels at <5.7%, and blood glucose levels at <140 mg/dl (<7.8 mM) 2 hrs. following an oral glucose challenge [36]. There are multiple tissues that coordinately regulate blood glucose levels, and the role of these tissues varies dependent on the fed or fasted state of the individual. In the fasted state, the liver maintains systemic glucose homeostasis via glycogenolysis and gluconeogenesis. In the fed state, glucose released from the digestive system into the bloodstream causes a rise in blood glucose levels that triggers an increase in insulin production and release from the β-cells of the pancreas. The rise in blood insulin levels triggers the following events: (1) inhibition of liver glycogenolysis, gluconeogenesis and glucose secretion; (2) stimulation of adipose tissue glucose uptake; and (3) stimulation of skeletal muscle glucose uptake. Thorough reviews on the regulation of systemic glucose homeostasis are provided in the following articles: [37,38,39].

### 4.2. Dysregulation in Type 2 Diabetes

Type 2 diabetes is a chronic metabolic disease characterized by a dysregulation of systemic glucose homeostasis. It is diagnosed when fasted blood glucose levels are >126 mg/dL (>7.0 mM), HbA1c levels are >6.5%, and blood glucose levels are >200 mg/dL (>11.1 mM) 2 hrs. following an oral glucose challenge [36]. While the exact causes of type 2 diabetes remain incompletely understood, numerous studies have linked impairments in key glucoregulatory functions in the pathogenesis of the disease. These include: (1) Impaired insulin-mediated inhibition of hepatic glucose output [40]; (2) impaired insulin-stimulated glucose uptake into adipose tissue [41,42]; (3) impaired release of insulin from the pancreas; and (4) impaired insulin-stimulated glucose uptake into skeletal muscle [43,44]. Thorough reviews on the tissues and physiological processes involved in the dysregulation of systemic glucose homeostasis in type 2 diabetes are provided in the following articles: [45,46,47].

### 4.3. Effects of Aerobic Training in Type 2 Diabetes

Aerobic exercise training is well-known for its ability to improve systemic glucose control in both individuals and animals with type 2 diabetes. Studies in both men and women with type 2 diabetes have demonstrated the following beneficial effects: (1) 8 weeks of aerobic walking (30 min/day, 3 days/week) decreased HbA1c levels ~18% [8]; (2) 12 weeks of bicycle training (60 min/day, 3 days/week) decreased fasted blood glucose levels ~14% [2]; (3) 16 weeks of cardiovascular machine-based training (60 min/day, 3 days/week at 60–65% max heart rate) decreased fasted blood glucose levels ~10% and HbA1c levels ~1% [48]; (4) 16 weeks of interval walking (3 min fast-3 min slow cycles; 60 min/session, 5 sessions/week) decreased fed plus fasted blood glucose levels ~8.5% [3]; (5) 24 weeks of aerobic walking, running, cycling or calisthenic exercises (60 min/day, 4 days/week at 60–70% max heart rate) decreased fasted blood glucose levels ~28% and HbA1c levels ~7% [49]; (6) 26 weeks of progressive treadmill running or cycle ergometer training (15–20 min/day, 3 days/week at 60% max heart rate, up to 45 min/day, 3 days/week at 75% max heart rate) decreased HbA1c levels ~7% [4]; (7) 36 weeks of treadmill walking decreased HbA1c levels ~0.5% in subjects with the most severe diabetes (HbA1c >7.0%) [1]; (8) 52 weeks of progressive treadmill running or cycle ergometer training (20 min/day, 2–3 days/week at 60% max heart rate up to 60 min/day, 2–3 days/week at 75% max heart rate) decreased HbA1c levels ~15% [50]; and (9) 8 years of aerobic machine training (90 min/day, 3 days/week at 50–80% VO_2_max) decreased HbA1c levels ~22% [51]. Similarly, studies in rodents demonstrated the following beneficial effects: (1) 7 weeks of motorized wheel exercise (1 hour/day, 5 days/week at 5.2 meters/min) decreased blood glucose levels ~12% in db/db mice compared to sedentary controls [6]; (2) 8 weeks of progressive treadmill running (8% grade, 18 m/min, 5 days/week, 40 min/day up to 120/day) decreased blood glucose levels ~20% at 15 and 30 min following an oral glucose challenge in Zucker fatty rats compared to sedentary controls [5]; (3) 12 weeks of voluntary wheel running decreased fasting blood glucose levels ~20% in diabetic db/db mice compared to sedentary controls [52]; (4) 12 weeks of swimming (1 hour/day, 3 days/week) decreased blood glucose levels ~11% and HbA1c levels ~7% in diabetic Zucker fatty rats compared to sedentary controls [53,54]; (5) 12 weeks of treadmill running (up to 17% incline, 10–15 meters/minute, 1 hour/day, 5 days/week) decreased fasting blood glucose ~14% in fatty Zucker rats compared to sedentary controls [7]; and (6) 13 weeks of swimming resulted in 60% lower fasted glucose, 52% lower fed glucose, as well as fatty Zucker rats were significantly more glucose tolerant than sedentary controls [55].

### 4.4. Effects of Resistance Training in Type 2 Diabetes

Resistance exercise training has been shown to improve systemic glucose control in both individuals and rodents with type 2 diabetes. Studies in both men and women with type 2 diabetes have demonstrated the following beneficial effects: (1) 8 weeks of progressive free weight and weight machine training involving arms and legs (2 days/week, 7 exercises/session, 3 sets of 10 repetitions at 60% 1 repetition max up to 100% of initial 1 repetition max) decreased HbA1c levels ~18% [8]; (2) 16 weeks of weight machine training involving arms and legs (3 days/week, 5 exercises/session, 8 repetitions at 60–80% max, up to 8 repetitions at 70–80% max) reduced HbA1c levels ~13% [9]; (3) 16 weeks of progressive free weight and weight machine training of arms and legs (3 days/week, 10 exercises/session, progressing from 3 to 6 sets/week of 10–15 max repetitions) decreased fasted blood glucose levels ~28% and HbA1c levels ~14% [56]; (4) 16 weeks of free weight and weight machine training of arms and legs (3 days/week, 7 exercises/session, 10 max repetitions) decreased fasted blood glucose levels ~7% and HbA1c levels ~5% [48]; (5) 24 weeks of progressive free weight and weight machine training of the arms, legs and abdomen (3 days/week, 9 exercises/session, 8–10 repetitions at 50–60% max progressing to 10 repetitions at 75–85% max) lowered HbA1c levels ~2% [10]; (6) 24 weeks of weight machine training of arms and legs (4 days/week, 8 exercises/session, 2–3 sets of 8–10 max repetitions) decreased fasted blood glucose levels ~9% and HbA1c levels ~3% [49]; (7) 24 weeks of weight machine (i.e., bioDensity™) training of arms and legs (1 day/week, 5–10 min/day) reduced fasted blood glucose levels ~11% and HbA1c levels ~8% in subjects with the most severe diabetes (HbA1c >7.5%) [57]; (8) 26 weeks of progressive machine weight training of arms, legs and abdomen (2 days progressing to 3 days/week, 7 exercises/session, 8–12 max repetitions) reduced HbA1c levels ~4% [4]; and (9) 52 weeks of progressive machine weight training of arms, legs and abdomen (10 exercises/session, 1 set of 1 max repetitions, 2 days/week up to 3 sets of 8–10 max repetitions, 3 days/week) decreased fasted blood glucose levels ~15% and HbA1c levels ~8% [50].

In addition, studies in rodents have demonstrated similar beneficial effects of resistance training on systemic glucose control. These studies found the following effects: (1) 7 weeks of progressive weighted ladder climbing (80° incline, 10 climbs/session, 5 sessions/week starting with an external load equal to 10% body weight and increasing up to 70% body weight) decreased fasted blood glucose levels ~30% in monosodium glutamate diet-induced diabetic rats [13]; (2) 8 weeks of progressive weighted ladder climbing (85° incline, 10 climbs/session, 3 sessions/week starting with an external load equal to 50% body weight and increasing up to 80% max load) reduced fasted blood glucose levels ~55% and improved glucose tolerance 50% in diabetic Zucker fatty rats compared to sedentary controls [11]; and (3) 10 weeks of isometric wire hang training (3 min/bout, 3 bouts/session, 5 sessions/week) decreased blood glucose levels ~30% 2 hrs. following an intraperitoneal glucose challenge in high fat diet-induced hyperglycemic C57BL/6N mice compared to sedentary controls [58].

## 5. Skeletal Muscle Glucose Transport

Skeletal muscle plays a critical role in maintaining blood glucose homeostasis. Studies in healthy individuals have demonstrated that in the post-prandial state that skeletal muscle is responsible for taking up 70–90% of the glucose from the blood [59,60]. The following sections review the current literature and highlight key gaps in our current understanding of the processes involved in the regulation of glucose transport in skeletal muscle as well as the ability of both aerobic and resistance exercise training to alter this process.

Skeletal muscle takes up glucose from the extracellular fluid into the cell via a surface membrane sugar transport protein [61]. There are two major families of sugar transport proteins found in mammalian cells: (1) The solute carrier family 2 (gene family SLC2) which consists of fourteen facilitative glucose transporters (GLUTs 1–14); and (2) the solute carrier family 5 (gene family SLC5) which consists of six sodium-dependent glucose co-transporters (SGLTs 1–6). These two families differ in their structural and functional characteristics. The GLUTs possess 12 transmembrane domains, an N-linked glycosylation motif [62,63], and transport sugars via facilitated diffusion; whereas, the SGLTs possess 14–15 transmembrane domains [64] and couple glucose with sodium transport to facilitate cellular glucose uptake [65]. In addition to these characteristics, the GLUTs and SGLTs can also vary greatly in their ability to transport different sugars, their subcellular localization, as well as their susceptibility to chemical inhibitors. For a thorough description of these characteristics, please see the following reviews on this topic [62,66,67,68]. Skeletal muscle expresses many sugar transporter isoforms, including: GLUT1, GLUT3, GLUT4, GLUT5, GLUT6, GLUT8, GLUT10, GLUT11, GLUT12, SGLT1, SGLT2, SGLT3, and SGLT4; and Table 1 provides a list of the different skeletal muscle models in which each of these sugar transporter isoforms has been observed.

### 5.1. Regulation of Basal Glucose Transport

GLUT1 is largely considered the GLUT isoform responsible for basal/non-insulin stimulated muscle glucose transport due to its localization predominantly on the muscle cell surface [76,77,108]. This postulation is consistent with muscle-specific GLUT1 overexpression mouse studies demonstrating a positive relationship between increasing GLUT1 levels and increases in basal muscle glucose transport [108,109]. However, additional scrutiny of these studies demonstrated that despite an ~40-fold increase in GLUT1 protein in the muscles from the overexpression mice, there was only an ~9-fold increase in basal muscle glucose transport [109]. This finding suggests that in skeletal muscle the mechanism regulating glucose transport via GLUT1 is more complex than just cell surface expression. Consistent with that interpretation, recent work in L6 myoblasts demonstrated that mutation of GLUT1 Ser490 to Ala490 decreased basal muscle glucose transport 44% with only a 17% reduction in cell surface localization [75]. Additional studies are needed utilizing muscle-specific GLUT1 null models to definitively assess the contribution of GLUT1 to basal skeletal muscle glucose uptake.

### 5.2. Regulation of Acute Insulin—And Exercise/Contraction-Stimulated Glucose Transport

GLUT4 is the most abundant glucose transporter isoform expressed in skeletal muscle [110]. Unlike GLUT1 which resides almost exclusively on the cell surface, in the basal (non-insulin stimulated) state, GLUT4 resides both on the cell surface (~20% of total GLUT4 protein) and in GLUT4 storage vesicles within the cell (~80% of total GLUT4 protein) [111,112]. In response to stimulation by insulin or exercise/muscle contraction, GLUT4 translocates from intracellular storage vesicles to the muscle cell surface where it plays an essential role in mediating acute insulin- and exercise/muscle contraction-stimulated muscle glucose transport [87]. Notably, skeletal muscle insulin- and contraction-stimulated GLUT4 translocation to the plasma membrane and t-tubules is additive suggesting potential distinct pools of GLUT4 [89,113,114]. The intracellular signaling and docking mechanisms by which GLUT4 translocation occurs has been and continues to be extensively studied. Thorough reviews on this topic can be found in the following papers: [111,115,116,117,118].

### 5.3. Dysregulation of Insulin-Stimulated Glucose Transport in Type 2 Diabetes

In individuals with type 2 diabetes, the ability of insulin to stimulate skeletal muscle glucose transport is impaired [119,120,121,122]. Studies in both human and rodents have demonstrated that this impairment in insulin-stimulated muscle glucose transport is due to a disruption in GLUT4 translocation to the muscle cell surface rather than an alteration in total muscle GLUT4 protein content [115,123]. While insulin-stimulated muscle GLUT4 translocation and glucose transport is impaired in type 2 diabetes, the ability of acute bouts of exercise or muscle contractile activity to stimulate GLUT4 translocation and glucose transport remains intact [124,125,126,127].

### 5.4. Regulation of Aerobic Exercise Training-Induced Glucose Transport

Aerobic exercise training has been shown to increase GLUT4 protein levels 20–70% in human [128,129,130,131,132,133,134,135,136] and rodent skeletal muscle [137,138,139,140,141], suggesting that aerobic training would enhance acute insulin- and exercise/muscle contraction-stimulated muscle glucose transport. Consistent with this prediction, studies have demonstrated that aerobic training enhances insulin-stimulated muscle glucose disposal 20–100% [131,134,142,143,144,145,146]. However, consistent with the aerobic training effect of increased reliance on lipid utilization, work in human vastus lateralis muscle has demonstrated that only 3 weeks of aerobic training decreases exercise-stimulated muscle GLUT4 translocation and glucose transport at a given workload [128]. Additional studies are needed examining the relationship between alterations in muscle glucose transport during exercise and changes in total muscle GLUT4 protein levels following a long-term (>6 weeks) aerobic training program.

**Figure 1:**
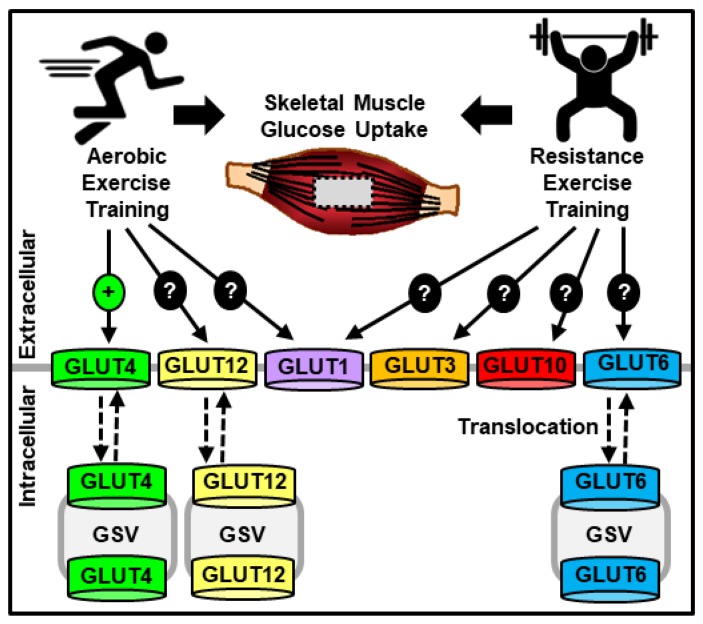
Model of aerobic and resistance exercise training effects on skeletal muscle glucose transporters (GLUTs). Legend: GSV = GLUT storage vesicle.

Alterations in the intracellular signaling mechanisms regulating GLUT4 translocation represent one possible explanation for how aerobic exercise training alters muscle glucose transport. An additional explanation is the involvement of other glucose transporter isoforms (Figure 1). Only a few studies have examined the effects of aerobic exercise training on GLUT isoforms other than GLUT4, and these studies examined GLUT1, GLUT5, GLUT8 and GLUT12. One study in humans did not see any alteration in muscle GLUT1 protein content following 6 weeks of a progressive cycling program (30 min at 70–75% max heart rate up to 50 min at 70–85% max heart rate) and strikingly saw a 72% decrease in GLUT5 protein levels [127]. In the muscle of endurance trained collegiate athletes, GLUT8 and GLUT12 mRNA levels did not differ from sedentary controls [147], but GLUT12 protein levels increased 104% in human vastus lateralis following 6 weeks of a progressive cycling program [129]. However, none of these studies completely include or exclude the potential involvement of GLUT1, GLUT8 or GLUT12 in this process. Since aerobic exercise training does not stimulate basal muscle glucose transport, any additional transporters involved in aerobic training-induced changes in muscle glucose transport would have to possess the ability to either alter their transport activity via post-translational modification and/or translocate to the muscle cell surface. Intriguingly, studies have demonstrated that GLUT1, GLUT8 and GLUT12 each possess at least one of these characteristics. As described above, while GLUT1 is localized predominantly on the muscle cell surface [76,77,108], its transport activity can also be regulated by phosphorylation on Ser490 [75] and Ser226 [148]. In contrast, both GLUT8 and GLUT12 contain an endocytic dileucine motif, and studies in 3T3L1 adipocytes or HEK293 cells have shown that mutation of this motif alters their cell surface localization [149,150]. Additional studies are needed to not only investigate the role of GLUT1, GLUT8 or GLUT12 in this process but also to assess whether other GLUT isoforms may be involved.

### 5.5. Regulation of Resistance Exercise Training-Induced Glucose Transport

Resistance exercise training increases glucose transport into skeletal muscle [151,152,153], and in rodent models it is clear that this increase in glucose uptake occurs independent of changes in muscle mass [152,153]. However, unlike aerobic exercise training that consistently increases muscle GLUT4 protein content, the ability of resistance exercise training to increase GLUT4 levels is less clear (Figure 1). While 6 weeks of intense progressive resistance training increased GLUT4 protein levels ~40% in the vastus lateralis of men with type 2 diabetes [151], the same training regimen failed to significantly alter muscle GLUT4 protein content in the healthy controls [151]. In addition, in mouse rectus femoris muscle, 10 weeks of isometric resistance exercise training increased GLUT4 protein levels ~70% [58]; yet, no change in GLUT4 protein levels was observed in rat gastrocnemius following 7 weeks of progressive weighted vertical ladder climbing [13]. The findings suggesting no role for GLUT4 in resistance training-induced muscle glucose transport are supported by recent work in muscle-specific GLUT4 knockout mice that demonstrated no impairment in plantaris muscle glucose transport following 5 days of functional overload, a model of resistance exercise training [73]. Collectively these results suggest that GLUT4 is not the sole mediator of resistance exercise training-induced increases in muscle glucose transport and propose that additional glucose transporter(s) play a role in this process.

While the identity of these additional glucose transporter(s) is currently unknown, studies have suggested a possible role for GLUT1, GLUT3, GLUT6, GLUT10 and/or SGLT3. In the vastus lateralis muscle of individuals with type 2 diabetes, 16 weeks of progressive resistance training increased SGLT3 mRNA and protein levels compared to sedentary controls [106]. However, work in Xenopus oocytes did not demonstrate any d-glucose transport by SGLT3 [107], and recent work in mouse plantaris muscle demonstrated no effect of the chemical SGLT inhibitor, phlorizin, on functional overload-induced muscle glucose transport [73]. Together these findings suggest that SGLTs are not necessary for loading-mediated muscle glucose transport. In the plantaris muscle of both wild-type and muscle-specific GLUT4 knockout mice, 5 days of functional overload increased the protein levels of GLUT1 ~150–300%, GLUT3 ~130%, GLUT6 ~250% and GLUT10 ~200–250% [73], suggesting a role for one or more of these GLUT isoforms in resistance training-induced muscle glucose transport. This finding is consistent with studies performed in cardiac, smooth or skeletal muscle that investigated these GLUT isoforms in the regulation of muscle cell growth, development, and redox buffering. These studies demonstrated: (1) An ~150% increase in GLUT1 protein levels following pressure overload in the heart [154]; (2) a transient but ~900% increase in GLUT3 mRNA levels during L6 myocyte fusion [83]; (3) an ~45% increase in GLUT3 protein levels in L6 myotubes following long-term insulin-like growth factor-1 exposure [81]; and (4) an increase in oxidative stress following loss of function mutations in GLUT10 arterial smooth muscle cells [155,156]. Future studies in muscle-specific GLUT knockout mouse models are needed to fully assess the role of any of these GLUT isoforms in the regulation of resistance training-induced muscle glucose transport.

## 6. Skeletal Muscle Glucose Metabolism

**Figure 2:**
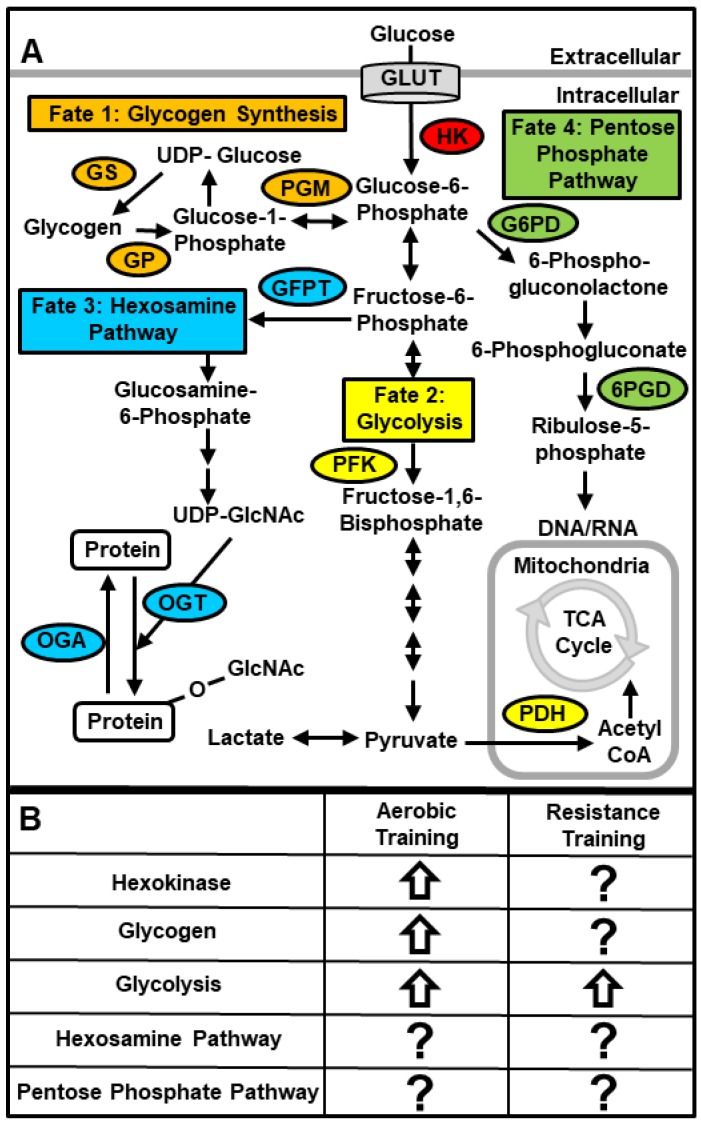
Major enzymes and metabolites of skeletal muscle glucose metabolism (**A**), and the effects of exercise training on the four major metabolic pathways (**B**). Key: 6PGD, 6-Phosphogluconate Dehydrogenase; G6PD, Glucose-6-Phosphate Dehydrogenase; GFPT, Glutamine-Fructose-6-Phosphate Transaminase; GP, Glycogen Phosphorylase; GS, Glycogen Synthase; HK, Hexokinase; OGA, O-GlcNAcase; OGT, O-GlcNAc Transferase; PFK, Phosphofructokinase; PGM, Phosphoglucomutase; PDH, Pyruvate Dehydrogenase.

Glucose transported into skeletal muscle is phosphorylated by hexokinase to form glucose-6-phosphate thereby trapping it in the cell. After this step there are four main cellular fates of glucose, and the partitioning of glucose into these metabolic pathways has critical consequences for future increases in muscle glucose transport and phosphorylation. The following sections review the current literature and highlight key gaps in our current understanding of the important enzymes and metabolites involved in the regulation of skeletal muscle glucose metabolism, as well as the ability of both aerobic and resistance exercise training to alter these metabolic pathways (Figure 2).

### 6.1. Hexokinase

Hexokinase is one of the most critical enzymes involved in skeletal muscle glucose metabolism, as the phosphorylation of glucose prevents it from diffusing back out of the cell. In resting mouse skeletal muscle, basal glucose uptake was not affected by an ~800% increase in hexokinase II protein levels demonstrating that hexokinase activity does not limit skeletal muscle glucose transport in the basal (non-insulin-stimulated) state [157]. Acute stimulation of skeletal muscle by insulin or exercise/contraction increases hexokinase activity in both human and rodent skeletal muscle [158,159,160]. In contrast to the basal state, hexokinase expression/activity regulates muscle glucose transport in response to insulin and exercise. In muscle-specific hexokinase II overexpression mice, muscle glucose transport in response to both hyperinsulinemia and an acute 10 min bout of treadmill running was increased ~30–40% [157], suggesting that under stimulated conditions hexokinase activity controls the amount of muscle glucose transport. Consistent with those findings, in mice with a 50% reduction of hexokinase activity, soleus muscle glucose transport was decreased ~70% following an acute 30 min bout of treadmill running [161]. However, in the gastrocnemius muscle of hexokinase knockdown mice neither insulin nor exercise-mediated glucose transport was altered [161,162]. Together these findings suggest that hexokinase can be a limiting factor to muscle glucose uptake, but only under conditions of extremely elevated muscle glucose transport.

Numerous studies have shown that aerobic exercise training increases hexokinase protein and activity levels ~25–100% in both human and rodent skeletal muscle [163,164,165,166,167,168,169,170,171,172]. In contrast, the effects of resistance exercise training on muscle hexokinase levels is less clear. In the skeletal muscle of healthy men, one study demonstrated a 28% increase in hexokinase activity following 10 weeks of isokinetic strength training [173]; a second study demonstrated no change in hexokinase activity following 12 weeks of high intensity resistance training [174]; and a third study found a ~40% decrease in hexokinase activity following 24 weeks of high intensity progressive resistance training [175].

### 6.2. Cellular Fates of Glucose in Skeletal Muscle

#### 6.2.1. Glycogen

Glycogen is the polysaccharide storage form of glucose in skeletal muscle. Glucose entering muscle is committed to storage as glycogen when glucose-6-phosphate is converted to glucose-1-phosphate by the enzyme, phosphoglucomutase. Further metabolism to UDP-glucose enables the enzyme glycogen synthase (GS) to generate the multi-branched glucose polymers that are characteristic of a glycogen particle. When cellular energy demands increase, glycogen can be degraded to glucose-1-phosphate by the enzyme glycogen phosphorylase (GP), and then ultimately metabolized via glycolysis to make adenosine triphosphate (ATP).

In non-stimulated skeletal muscle, glycogen levels are determined by the balance between glycogen synthesis and glycogen degradation. Consistent with this statement, in muscle-specific GS overexpression mice muscle glycogen levels are increased ~400% [176]; while in muscle-specific GS1 knockout mice muscle glycogen levels are decreased 65% in the fasted state [177]. 

Aerobic exercise training is well-known to increase glycogen levels and glycogen synthesis rates in both human and rodent skeletal muscle. These studies demonstrated the following results: (1) 6 weeks of stair climb training (4 days/week, 45 min/day at 65% VO_2_max) increased muscle glycogen synthesis rates ~100% [171,178,179]; (2) 7 weeks of voluntary wheel running increased glycogen levels ~30% in triceps muscles from female Sprague Dawley rats [179]; (3) 10 weeks of progressive high intensity cycle ergometry training (3 days/week, 90–100% VO_2_max, 4 × 5 min bouts up to 5 × 5 min bouts) and progressive running (3 days/week at 30 min/day up to 40 min/day) increased glycogen levels ~80% in vastus lateralis muscle [132]; (4) 12 weeks of indoor cycle training (60 min/day, 4 days/week at 75–90% max heart rate) increased glycogen levels ~80% in vastus lateralis muscle [171,178,179]; (5) 20 weeks of cycle ergometer training (1 hour/day, 4 days/week at 75–90% VO_2_max) increased muscle glycogen levels ~150% [180]; and (6) endurance trained cyclists have ~65% higher muscle glycogen content than untrained individuals 48–72 hours after an exhaustive cycling bout [181]. Thus, together these results suggest that aerobic training-induced increases in muscle glycogen content occur to provide a greater capacity to fuel future muscle contractions [182,183]. 

Unlike aerobic training, the effects of resistance exercise training on muscle glycogen levels are more variable. In both men and women, the following effects have been reported: (1) 6 weeks of progressive free weight and weight machine training of the upper body (3 days/week, 4 exercises/day, 10 sets/week up to 32 sets/week of 10 repetitions/set at 60% of 1 repetition max for each exercise) did not alter glycogen content [184,185]; (2) 6 weeks of progressive weight machine training of the leg (3 days/week, 3 exercises/session, 10 repetitions at 50% of 1 repetition max up to 8–12 repetitions at 70–80% max) increased muscle glycogen levels ~14% [151]; (3) 16 weeks of weight machine training involving arms and legs (3 days/week, 5 exercises/session, 8 repetitions progressing from 60–80% to 70–80% max) increased muscle glycogen levels ~30% [9]; (4) 16 weeks of progressive lower body pneumatic training (3 days/week, 2 exercises/session, progressing from 60–65% to 75–80% of 1 repetition max up) increased muscle glycogen levels ~45% [9,106]; and (5) 20 weeks of resistance exercise training (2–3 days/week, 4 exercises/session, 3–5 sets/day of 8–10 repetitions) increased glycogen levels ~20% in triceps brachii muscles [186]. In addition, the following results were demonstrated in rodent skeletal muscle after resistance exercise training: (1) 12 weeks of squat training (3 days/week, 10 repetitions at 75% of one repetition max) increased gastrocnemius muscle glycogen levels 40–50% in male Sprague-Dawley rats [152]; (2) 12 weeks of progressive weighted ladder climbing (80° incline, 4–8 climbs/day, 3 days/week with a load of 75% body weight up to 100% body weight) increased muscle glycogen levels 950–3500% in female Wistar rats [12]; (3) 12 weeks of progressive weighted ladder climbing (80° incline, 3–6 climbs/day, 4 days/week with a load of 10% body weight up to 200% body weight) increased muscle glycogen levels 20–45% in male Wistar rats [12,14]; and (4) 4 days of functional overload did not change soleus muscle glycogen levels in male Swiss albino mice [12]. Thus, taken together these data suggest that the type, duration, and intensity of the resistance training program are important factors in determining the effects of resistance training on skeletal muscle glycogen content.

#### 6.2.2. Glycolytic Flux

Glucose transported into muscle enters glycolysis once fructose-6-phosphate is converted to fructose-1,6-bisphosphate by the enzyme phosphofructokinase (PFK). Fructose-1,6-bisphosphate then undergoes the multi-step, sequential conversion to pyruvate. Muscle pyruvate has two main fates: (1) reduction to lactate; or (2) oxidation to acetyl-CoA by pyruvate dehydrogenase (PDH), which is then further metabolized via the tricarboxylic acid (TCA) cycle. Complete oxidation of glucose through the TCA cycle and mitochondrial electron transport chain yields 36 molecules of ATP.

Glucose flux through glycolysis plays a critical role in regulating skeletal muscle contractile function. In humans, genetic loss of muscle PFK activity (known as Tarui disease or glycogen storage disease type 7) is characterized by increases in skeletal muscle glucose-6-phosphate levels (~360–1740%), fructose-6-phosphate levels (~280–1500%), and muscle glycogen levels (~75–350%) in the resting state; along with impairments in exercise tolerance (i.e., shorter time to fatigue) [187]. This clinical profile is mimicked in muscle PFK knockout mice which increased muscle glucose-6-phosphate levels (~320%) and glycogen levels (~110%) at rest; along with decreased ATP levels (~50%) and a severely shortened time to fatigue (<1.5 min) when subjected to treadmill running [188]. Impairments in exercise endurance capacity were also observed in skeletal muscle specific-PDHα1 knockout mice [189], highlighting the importance of the ATP generated from complete glucose oxidation in muscle contraction and whole-body locomotion.

Aerobic exercise training results in variable changes in skeletal muscle glycolytic capacity. This is demonstrated in studies conducted in both humans and rodents that showed 0–120% increases in muscle PFK activity following aerobic training. These studies found: (1) 20 weeks of cycle ergometer training (4 days/week, 1 hour/day at 75–90% VO_2_max) increased PFK activity ~120% in human vastus lateralis muscle [180](2) 6 weeks of treadmill running (5 days/week, 6 bouts of 4.5 min at 40 m/min) increased PFK activity 20–25% in rat soleus and deep vastus lateralis muscle, but not in the superficial vastus lateralis or the diaphragm [190]; and (3) 16 weeks of voluntary wheel cage running increased PFK activity ~87% in rat white gastrocnemius muscle [191], but did not alter it in the soleus, plantaris or red gastrocnemius [191]. In contrast, studies have demonstrated that aerobic training increases glucose oxidative capacity, as evidenced by the following findings: (1) 6 weeks of high intensity interval training (3 days/week, 10 × 4 min intervals/day at ~90% VO_2_max,) increased PDH activity ~20% in human vastus lateralis muscle [136]; and that (2) 8 weeks of cycle ergometer training (5 days/week, 1 hour/day at 75% VO_2_max) increased PDH activity ~30% in human vastus lateralis [192].

Similar to aerobic training, resistance exercise training induces changes in skeletal muscle that favor an increase in the capacity of glucose flux through glycolysis. Studies performed in humans and rodents demonstrated the following findings: (1) 14 weeks of progressive free weight training of arms and shoulders (3 days/week, 3 exercises/session, 3 sets/exercise with increasing loads) increased PFK activity ~20% in human deltoid muscle [193]; (2) 24 weeks of progressive free weight squatting and jumping training did not alter PFK activity in human vastus lateralis muscle [175]; and (3) 10 weeks of isometric wire hang training (5 sessions/week, three 3-minute bouts/session) increased rectus femoris muscle PFK mRNA levels ~320% in high fat diet-induced hyperglycemic C57BL/6N mice compared to sedentary controls [58]. In addition, in mouse soleus muscle, 4 days of functional overload increased 3-[^3^H]-D-glucose conversion ^3^H_2_O ~50% [17], a process that occurs during the enolase reaction. Thus, collectively, these findings suggest that both aerobic and resistance training increase the capacity of skeletal muscle to utilize glucose through glycolysis to generate ATP.

#### 6.2.3. Hexosamine Pathway

The hexosamine pathway is a glucose utilizing pathway that is initiated when fructose-6-phosphate is converted to glucosamine-6-phosphate by the enzyme glutamine fructose-6-phosphate transaminase 1 (GFPT1). The hexosamine pathway produces UDP-N-acetylglucosamine and other nucleotide hexosamines which are used for the glycosylation, N-linked GlcNAcylation, and O-linked GlcNAcylation of proteins [reviewed in [194]]. Protein O-GlcNAcylation is one of the most commonly studied modifications of hexosamine pathway activity and is controlled by the following two enzymes: (1) O-GlcNAc transferase (OGT), which adds O-GlcNAc to proteins; and (2) O-GlcNAcase (OGA), which removes O-GlcNAc from proteins.

Multiple studies in both human and rodent muscle have linked increased hexosamine pathway activity to the development of muscle insulin resistance. In the vastus lateralis muscle of individuals with type 2 diabetes, O-GlcNAcylated protein levels were ~50% higher compared to lean, healthy controls [195]. In addition, while transgenic mice overexpressing GFPT1 exhibited a ~50% reduction in insulin-stimulated muscle glucose disposal [196], muscle-specific OGT knockout mice exhibited enhanced insulin-stimulated muscle glucose transport [195]. Thus, collectively these studies demonstrate a direct positive relationship between activation of the hexosamine pathway and skeletal muscle insulin resistance.

Since aerobic and resistance exercise training are associated with enhancements in skeletal muscle insulin sensitivity and glucose transport, it could be postulated that exercise training would decrease hexosamine pathway activity. To date, only two studies have directly examined the effects of exercise training on the hexosamine pathway in skeletal muscle, and both examined the effects of aerobic training. In the vastus lateralis of postmenopausal women, one year of progressive plyometric training did not alter the mRNA levels of OGT or OGA compared to sedentary postmenopausal controls [197]. In contrast, six weeks of progressive treadmill running increased protein O-GlcNAcylation levels ~80–100% in both the soleus and extensor digitorum longus muscles of male Sprague Dawley rats [198]. Taken together these results suggest that exercise training-mediated adaptations in the hexosamine pathway and protein O-GlcNAcylation levels in skeletal muscle may be gender and/or species specific. However, given the conflicting findings and limited amount of studies investigating this interaction, any current conclusions should be considered with caution. Additional studies are needed in humans and rodent models from both sexes to fully assess the possible role of the hexosamine pathway in training-induced alterations in muscle glucose transport and metabolism.

#### 6.2.4. Pentose Phosphate Pathway

The pentose phosphate pathway is a glucose utilizing pathway that is initiated when glucose-6-phosphate is converted to 6-phosphogluconolactone by glucose-6-phosphate dehydrogenase (G6PD). It is used to make metabolites critical for skeletal muscle anabolism, including: (1) nicotinamide adenine dinucleotide phosphate (NADPH) for reductive biosynthesis reactions such as lipogenesis; (2) ribose 5-phosphate for nucleotide synthesis; and (3) erythrose-4-phosphate for aromatic amino acid synthesis. A second important enzyme in this pathway, 6-phosphogluconate dehydrogenase (6PGD), is responsible for the production of ribulose 5-phosphate from 6-phosphogluconate, and its activity is often measured to assess pentose phosphate pathway activity [199,200,201].

In non-stimulated skeletal muscle, the activity of the pentose phosphate pathway is low compared to most other tissues [202,203]. This finding is perhaps not surprising since skeletal muscle is a differentiated cell type, and at rest does not have the biosynthetic demands of proliferative cell types such as the liver. In contrast, studies have demonstrated an increase in the activity of the pentose phosphate pathway in skeletal muscle in response to damage/regeneration. In individuals with Duchenne’s muscular dystrophy, a condition characterized by a cycle of skeletal muscle degeneration and regeneration, muscle G6PD activity is increased ~400% and 6PGD activity ~300% compared to healthy age-matched controls [199]. In rat skeletal muscle, administration of the myotoxic agent Marcaine stimulated G6PD activity ~350% and 6PGD activity ~140% [200], while a muscle damaging bout of downhill running increased G6PD activity ~100–350% [204]. In addition, an acute bout of 10 min of high intensity tetanic contractions increased rat muscle ribose-5-phosphate levels [205]. Collectively these findings suggest that activation of the pentose phosphate pathway occurs in skeletal muscle to provide substrates for muscle repair processes.

The role of the pentose phosphate pathway in mediating either aerobic exercise training-induced or resistance exercise training-induced adaptations in skeletal muscle glucose metabolism has not yet been investigated. However, recent work utilizing transgenic mice expressing key signaling proteins involved in mediating exercise training-induced adaptations in muscle, such as the peroxisome proliferator-activated receptor gamma coactivator 1-alpha (PGC-1α) and Akt isoform 1 (Akt1), suggest an involvement of the pentose phosphate pathway in this process. PGC-1α is a transcriptional co-activator found in skeletal muscle that plays a critical role in mediating aerobic exercise training-induced increases in mitochondrial biogenesis, substrate metabolism, and fiber type switching [reviewed in [206]. Intriguingly, in skeletal muscle of muscle-specific PGC-1α overexpression mice there is an increase in key pentose phosphate pathway metabolites, including: 6-phosphogluconate, ribulose-5-phosphate, ribose-5-phosphate, NADPH, and sedoheptulose-7-phosphate (207]). Akt1 is a kinase found in skeletal muscle that plays a critical role in mediating resistance exercise training-induced muscle hypertrophic growth and protein synthesis (reviewed in [208,209]). In skeletal muscle of muscle-specific Akt1 overexpression mice there is also an increase in key pentose phosphate pathway metabolites and enzymes, including: ribose-5-phosphate, G6PD and 6PGD [201]. While taken together these results suggest that exercise training may stimulate glucose flux via the pentose phosphate pathway in skeletal muscle, additional studies examining skeletal muscle from exercise trained humans or rodents are needed to fully assess a role for this metabolic pathway in exercise training-induced adaptations in skeletal muscle glucose metabolism.

## 7. Conclusions

Both aerobic and resistance exercise training are beneficial in ameliorating the hyperglycemia associated with the metabolic disease, type 2 diabetes. This beneficial blood glucose lowering effect can be at least partially attributed to training-stimulated alterations in skeletal muscle glucose transport and glucose metabolism. This review of the current literature found that the effects of aerobic training are often larger in magnitude than those elicited by resistance training, and we speculate that this difference can be attributed to one or more of the following factors: (1) Duration of the training program; (2) intensity of the training; (3) prior training experience; (4) specific skeletal muscle examined; and/or (5) number of muscle groups stimulated by the exercise. In addition, throughout this review a number of key gaps in our current understanding of how both aerobic and resistance training alter skeletal muscle glucose transport and metabolism were identified. These key gaps included: (1) Mechanism underlying decreased exercise/contraction-stimulated glucose transport following aerobic training; (2) identity of the glucose transporter isoform(s) involved in mediating resistance training-stimulated muscle glucose transport; and (3) the exact proportion of glucose that enters each cellular fate in skeletal muscle in response to aerobic and resistance training. Future endeavors focused on determining the molecular and cellular factors that are responsible for the ability of exercise training to elicit beneficial effects on systemic glucose homeostasis, skeletal muscle glucose transport and/or skeletal muscle glucose metabolism should seek to fill in these critical knowledge gaps.

## Figures and Tables

**Table 1:** Skeletal muscle sugar transporters. Members of the facilitated glucose transporters (GLUT) and sodium-dependent glucose cotransporter (SGLT) families observed in human and rodent skeletal muscle.

Transporter	Gene	Muscle Models	References
**GLUT1**	SLC2A1	Human muscle	[69,70]
		C2C12	[71,72]
		Mouse muscle	[73,74]
		L6 myotubes	[72,75]
		Rat muscle	[76,77]
**GLUT3**	SLC2A3	Human muscle	[78,79]
		C2C12	[71]
		Mouse muscle	[73,80]
		L6 myotubes	[81,82]
		Rat muscle	[83,84]
**GLUT4**	SLC2A4	Human muscle	[85,86]
		C2C12	[71,72]
		Mouse muscle	[87,88]
		L6 myotubes	[82]
		Rat muscle	[76,89]
**GLUT5**	SLC2A5	Human muscle	[90,91]
		C2C12	[71]
		Mouse muscle	[92]
		L6 myotubes	[93]
		Rat muscle	[94]
**GLUT6**	SLC2A6	C2C12	[71]
		Mouse muscle	[73,95]
**GLUT8**	SLC2A8	Human muscle	[96]
		Mouse muscle	[95]
**GLUT10**	SLC2A10	Human muscle	[97]
		Mouse muscle	[73,95]
**GLUT11**	SLC2A11	Human vastus lateralis	[98]
		(slow-twitch fibers)	
**GLUT12**	SLC2A12	Human muscle	[99,100]
		C2C12	[71,101]
		Mouse muscle	[102]
		Rat muscle	[103]
**SGLT1**	SLC5A1	Human muscle	[104]
		Mouse muscle	[105]
**SGLT2**	SLC5A2	Mouse muscle	[105]
**SGLT3**	SLC5A4	Human muscle	[106]
		Mouse muscle	[107]
**SGLT4**	SLC5A9	Human muscle	[104]
